# User experience with two computerized cognitive intervention programs for people with mild cognitive impairment

**DOI:** 10.1186/s12877-025-06767-y

**Published:** 2025-12-10

**Authors:** Julia-Sophia Scheuermann, Elmar Graessel, Martin Schmitt, Petra Scheerbaum

**Affiliations:** Center for Health Services Research in Medicine, Department of Psychiatry and Psychotherapy, Uniklinikum Erlangen, Friedrich-Alexander-Universität Erlangen-Nürnberg (FAU), Schwabachanlage 6, Erlangen, D-91054 Germany

**Keywords:** User experience questionnaire, User experience, Mild cognitive impairment, Computerized cognitive training

## Abstract

**Background:**

Computerized cognitive training (CCT) is effective for people with mild cognitive impairment (MCI). But CCT must be “user friendly” and easy to handle for this target group.

**Objective:**

The aim of this study was to evaluate in a secondary data analysis the user experience with two CCT programs developed for people with MCI.

**Methods:**

In the framework of the randomized controlled study BrainFit-Nutrition (*N* = 270), two CCTs were examined: an individualized CCT (iCCT) with a machine learning system and more exercises versus a basic CCT (bCCT) with less exercises and without machine learning: both containing a set of cognitive training tasks. The User Experience Questionnaire (UEQ) was used to evaluate user satisfaction with the two CCTs at t6-follow-up (*n*_*UX*_=217; *n*_*UX−iCCT*_=109; *n*_*UX−bCCT*_=108). In addition to the benchmark, we examined, first, the differences between the CCTs; second, the correlation between self-reported training intensity and user satisfaction; and third, age and gender differences in the use of the CCTs.

**Results:**

For both CCT programs, the benchmark was above average or good for all scales. There were no significant differences between the CCT programs in 5 of the 6 UEQ scales. Only the Perspicuity scale showed significantly higher values for the bCCT than for the iCCT. Training intensity was positively correlated with the UEQ aspects Attractiveness, and hedonic quality (i.e. the scales Stimulation and Novelty) for each CCT program.

**Conclusion:**

Both CCT programs are user-friendly tools for individuals with MCI aged 60 years and older. Participants who used one of the CCT programs more frequently were more satisfied with the hedonic quality of the CCT program, whereby the training intensity of the iCCT was descriptively higher than in the bCCT. The results are very important, due to the rising number of persons with MCI and the need to offer various non-pharmacological intervention, that can be offered at a low threshold.

**Trial registration:**

ISRCTN10560738; registered November 23, 2021.

**Supplementary Information:**

The online version contains supplementary material available at 10.1186/s12877-025-06767-y.

## Introduction

Mild cognitive impairment (MCI) represents a transition between normal aging and dementia symptoms [1]. The prevalence of MCI increases with age. It affects 6.7% of 60-to-64-year-olds and one fourth of 80-to-84-year-olds [2]. When compared with cognitively healthy people, individuals with MCI have a higher risk of dementia conversion [3–5]. The 5-year transition rate to dementia concerns almost three in four individuals with amnestic MCI (72%; [6]). Consequently, interventions to prevent dementia should be conducted at the MCI stage.

To date there are no effective pharmacological interventions for the treatment of MCI [2, 7]. On a non-pharmacological level, however, there are already some treatment recommendations for decelerating the transition from MCI to dementia. These therapy recommendations primarily involve cognitive training, physical activity, and social activity [7–11].

Cognitive training is therefore a significant modifiable risk factor for MCI [8, 9, 12–15]. To improve cognitive abilities, multiple weekly training sessions in various cognitive domains are needed [8]. Computerized cognitive training (CCT) is now being used, as it offers the possibility of providing real-time feedback and more individualized training compared to classic, analogue cognitive training [16]. As a result, there are positive cognitive effects for people with cognitive impairments or dementia symptoms but also for cognitively healthy older people [17]. These positive effects can be increased through the use of machine learning within the CCT, as this offers the possibility to tailor the CCT to the different needs and individual cognitive performance. In consequence, participants can experience multi-domain benefits, improved motivation and an improvement in neuroplasticity [18].

In order to ensure that people can benefit from CCTs, it is important for the programs to be user-friendly and appropriate for the respective users’ levels of knowledge and abilities [19]. Usability considers the degree to which the program can be used effectively and to the satisfaction of the user [20]. Finally, user experience (UX) extends usability [19–21] and comprises the aspects of perception, emotion, beliefs, preferences, and behavior while and after using the program [21]. In particular, it should not be ignored that the current generation of people aged 60 years and older is characterized as digital immigrants. Measuring the UX and usability of cognitive intervention technologies can contribute to tailoring technologies to provide a good fit for the needs and characteristics of older people with MCI or dementia [19].

Previous studies on UX in older people with cognitive impairment have shown that they accept and use CCT programs regularly [19]. The CCT programs have a high level of acceptance [22], indicating that elderly people with cognitive impairment enjoy using the programs [22–24]. In addition, people using CCT programs are generally satisfied with the CCT programs [25, 26]. Manera et al. [26] showed that satisfaction with CCT programs is higher in people with Alzheimer’s dementia than in people with MCI. In addition, people with cognitive impairment are also intrinsically motivated to use CCT programs [26]. The reasons given for intrinsic motivation are to promote cognitive health [22, 27], avoid further cognitive decline [28], and avoid loneliness by participating in group settings [28]. Nevertheless, people with cognitive impairment have also expressed competence problems when using the technology [22, 29], and participants in González-Abraldes et al. [30] also had problems with the user interface of the CCT program.

Thus, CCT programs have already been shown to be effective for improving cognitive functioning in older people—with and without cognitive impairment [31, 32]. In the BrainFit-Nutrition study [33], CCT programs were developed on the basis of a pilot study [34]. Inappropriate technical devices can increase the negative feelings experienced by people with MCI, such as frustration and lack of self-confidence due to cognitive impairment [19, 35]. Subsequently, people with cognitive impairment might stop participating in the programs regularly or might drop out completely [32]. Wargnier et al. [36] showed that most programs are not adapted for older people with cognitive impairment. Therefore, UX is an important aspect when considering new training programs for people with cognitive impairment.

Consequently, we conducted the present study to meet four goals: first, to examine the UX of the two CCT programs — individualized CCT (iCCT) and basic CCT (bCCT) — from the BrainFit-Nutrition study specifically developed for people with MCI; second, to compare the UX of the two CCT programs; third, to analyze the relationship between user satisfaction and self-reported training intensity; and fourth, to examine gender and age differences in UX for both CCT programs.

## Methods

### Study design and sample

We analyzed the 6-month follow-up data from the randomized controlled trial BrainFit-Nutrition. The design of the intervention study included a prospective 2 × 2 randomized controlled trial consisting of two intervention arms: CCT and nutritional counseling. In both arms, we developed an active intervention specialized for people with MCI and an active control measure. Thus, the recruited participants - individuals with MCI - were randomly allocated to one of four intervention groups, stratified by age, gender, and the Montreal Cognitive Assessment (MoCA) baseline score. For the iCCT, the specialized intervention was an individualized self-learning system. As an active control measure, a bCCT was used. The study design was already published as a study protocol [33].

**Fig. 1:**
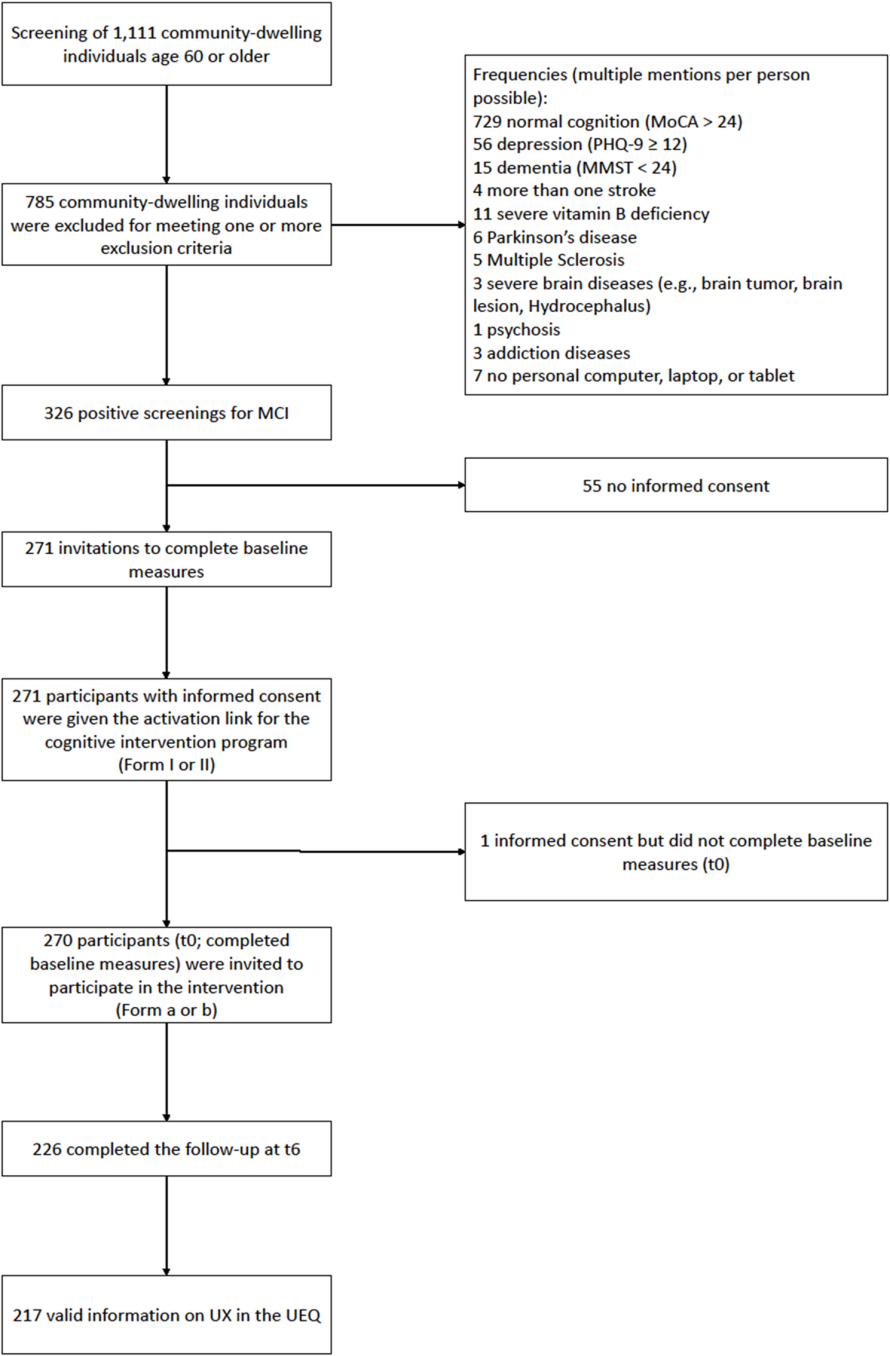
Flowchart of the study *Note*. Intervention form I/II comprises the two different kinds of computerized cognitive training (iCCT/bCCT); Intervention form a/b comprises the two kinds of nutrition counselling intervention presented in the BrainFit-Nutrition study. MCI: mild cognitive impairment; MoCA: Montreal Cognitive Assessment; MMST: Mini-Mental-Status Test; PHQ-9: Patient-Health Questionnaire; UEQ: User-Experience Questionnaire; UX: User Experience

In the BrainFit-Nutrition study, 1,111 people aged 60 and older in Germany were screened for MCI. The primary inclusion criterion for identifying MCI was a MoCA score ≤ 24 points and a MMSE score ≥ 24 points, which identified 326 individuals with MCI. Of these, 271 individuals with MCI agreed to participate and were randomized (based on age, gender, and MoCA). A total of *N* = 270 people completed the baseline survey in the form of a telephone interview. In the present analysis, we evaluated data from 217 people with complete information on UX (see Fig. 1).

All procedures were approved by the ethics committee of the Medical Faculty of the Friedrich-Alexander-Universität Erlangen-Nürnberg (Ref.: 21–318_1-B). A prospective registration was carried out by the International Standard Randomized Controlled Trial Number Registry (ISRCTN 10560738).

### Computerized cognitive trainings

As mentioned above, in the BrainFit-Nutrition study, two CCT programs were developed. Both are available for Windows, MacOS, and Linux PC or laptop and Android tablet. Participants were free to choose their input method. During the intervention period participants were recommended to use their CCT program three times per week for at least 30 min each time. As part of voluntary uploads, the participants were able to transmit the data from the exercise program to the university hospital. In the case of technical problems during the intervention phase, the study team and technical support were available to answer questions and provide assistance.

Both CCTs contain a set of cognitive tests for multiple domains, particularly memory span, processing speed, short term memory and executive functions. Sample images of both intervention programs are shown in supplement material 1.

### Individualized computerized cognitive training (iCCT; MCI specified intervention)

**Fig. 2:**
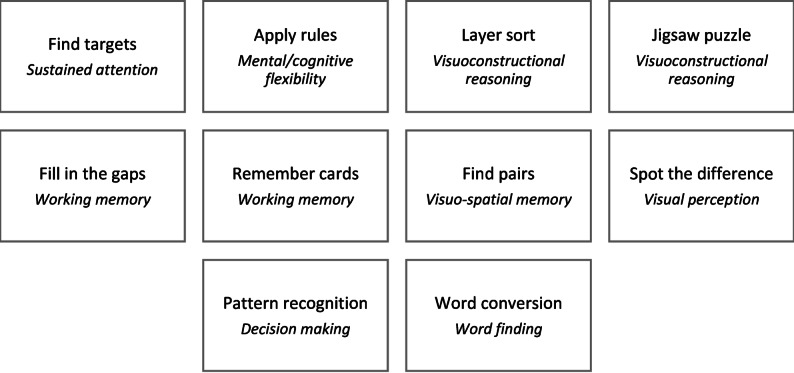
Tasks of the iCCT *Note*. The key domain of each task is shown in italics

The iCCT was based on an adaptive system, a type of machine learning and selected the exercises on the basis of the expected performance level of people with MCI [33, 34, 37]. The adaptive system comprises multiple logistic regression classifiers - one for each exercise and difficulty level - which predict the likelihood that an exercise will be successfully completed at a classifier’s level of difficulty, using a cut-off of a 65% success rate. Further, cognitive status based on the CCTB is used as input for the classifiers. To adapt to the participant, the results of the completed exercises are included in the training data of the system so that the classifier is retrained. Based on the CCTB-parameter cognitive status, classifiers are continuously trained. Thus, individualized training is possible. They train the basic parameters of information processing as well as short-term memory and require different types of decision-making (see Fig. 2). There are different levels of difficulty for each of the 10 iCCT exercises. The initial difficulty level of the exercises was determined by a machine learning system, which eliminates individual compensation strategies and generates the ideal level of difficulty for training each participant. To choose the entry level, the application selects the highest level of difficulty that the participant is likely to solve. The machine learning system relies on collected usage data as well as the cognitive test results. The iCCT thus aims to enhance the positive effects of a CCT program by providing exercises at the difficulty level that is most suitable for each participant.

### Basic computerized cognitive training (bCCT; active control program)

**Fig. 3:**
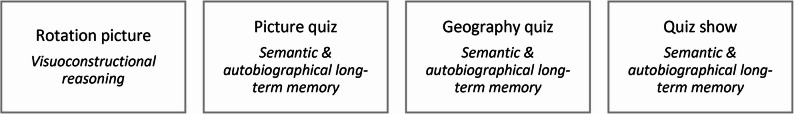
Tasks of the bCCT *Note*. The key domain of each task is shown in italics

The bCCT was based on exercises involving quizzes and visual tasks (see Fig. 3). They were designed to be playful and required simple strategies and long-term memory, among other things. Two of the four exercises were available in only one level of difficulty. The initial difficulty of the other exercises was based solely on the participant’s success with the previous exercise. The bCCT exercises were aimed at providing the active control group with an enjoyable computerized leisure activity with a limited number of cognitive tasks.

### Instruments

Sociodemographic data were collected, comprising age, gender, employment status, living situation, and highest educational level. Participants’ age was dichotomized into two categories with the median split method: 60 to 69 years, 70 years and older. Employment status was measured by asking whether the person was still working (yes/no). For the living situation, we identified whether the person was living alone (yes/no). Education was measured in accordance with the International Standard Classification of Education (ISCED; [38]). Training intensity was recorded in minutes per week (on average) as a self-report.

The User Experience Questionnaire (UEQ; https://www.ueq-online.org/) [39] was used to assess the user-friendliness of the two types of CCT program at the end of the intervention period. The UEQ is a standardized and validated instrument for measuring UX and user satisfaction with a digital application. It measures the UX scales of Attractiveness (6 items), Perspicuity (4 items), Efficiency (4 items), Dependability (4 items), Stimulation (4 items), and Novelty (4 items) across 26 items using bipolar adjective pairs. Moreover, Perspicuity, Efficiency, and Dependability are pragmatic quality aspects (goal-directed), while Stimulation and Novelty are hedonic quality aspects (not goal-directed). The participants indicated their assessment of the CCT program they used on a 7-point scale, weighted from + 3 to −3. Benchmark values [40] were used to classify the CCTs’ results into five categories: excellent (better than 90% of the evaluation results), good (better than 75% of the evaluation results), above average (better than 50% of the evaluation results), below average (worse than 50% of the evaluation results), or poor (worse than 25% of the evaluation results). This questionnaire has already been used in numerous studies involving individuals with cognitive impairments to determine the user experience of a technical program [19, 37, 41].

### Statistical analysis

The analysis was based on *N* = 217 participants with UX data. There were no missing values in this sample. To analyze the UX of the two CCT programs, first, input method (e.g. touch pad) and training intensity were analyzed descriptively. Using the UEQ benchmark, UX was analyzed for iCCT and bCCT. Therefore, the reliability of the UEQ scales was calculated for each of the two CCT programs (Cronbach’s α). The scales with a reliability below α < 0.6 [42] are highlighted in the analysis because such results should be interpreted with caution.

Group differences between the two CCT programs were analyzed with independent-samples *t* tests. In addition, the correlation between self-reported training intensity and user satisfaction in the UEQ was analyzed with a Pearson correlation. Finally, gender differences and age differences were examined within the CCT programs as part of two sensitivity analyses with median dichotomization. Statistical analyses were performed with IBM SPSS (V.28) at the 5% significance level.

## Results

### Sample characteristics

In the sample of 217 participants, 114 were female and 103 male. They were 71 years old on average. Regarding education, 77.7% of the men had a high educational level. In women, 43.9% had a high educational level. Almost a third of the participants were still working (31.6% of women and 27.2% of men). A gender difference occurred for the living situation. Whereas 45.6% of the female participants lived alone, only 14.6% of the male participants did. Participants used the following input methods when engaging in the CCT program: mouse and keyboard (76.0%), touch pad (15.7%), touch screen (7.8%), other (0.5%). Descriptively the iCCT was more often used than the bCCT (111 min/week vs. 105 min/week).

### Benchmark user experience

The reliability (Cronbach’s α) was α < 0.6 for two of the six scales — Efficiency and Dependability — for both CCT programs (see Supplemental Material 2). Thus, these two scales should be interpreted with caution.

**Fig. 4:**
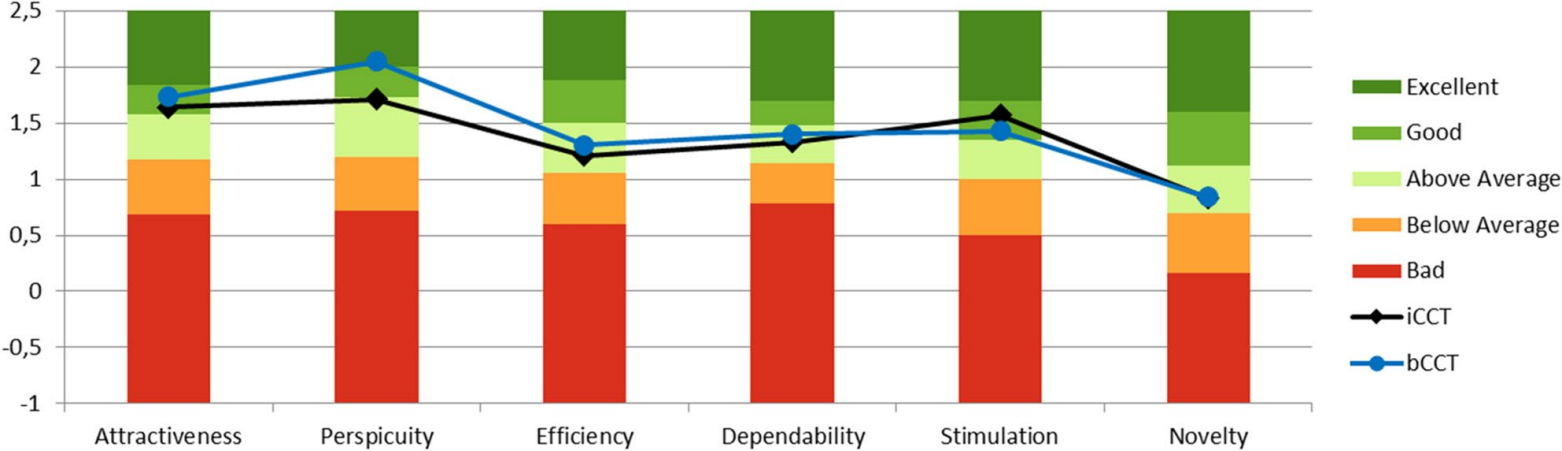
Benchmarks for the iCCT and bCCT *Note*. iCCT: individualized computerized cognitive training; bCCT: basic computerized cognitive training; The six UX dimensions can be assigned to the following three scales: Attractiveness (Attractiveness); pragmatic quality (Perspicuity, Efficiency, and Dependability; goal-directed); hedonic quality (Stimulation and Novelty; not goal-directed)

The iCCT (*n* = 109) was rated good in terms of Attractiveness (*M* = 1.64; *SD* = 0.95) and Stimulation (*M* = 1.57; *SD* = 0.98) (see Fig. 4). The Perspicuity (*M* = 1.71; *SD* = 0.90), Efficiency (*M* = 1.21; *SD* = 0.93), Dependability (*M* = 1.33; *SD* = 0.88), and Novelty (*M* = 0.83; *SD* = 1.15) scales were rated above average.

The bCCT (*n* = 108) also received good ratings for the Attractiveness (*M* = 1.73; *SD* = 1.10) and Stimulation (*M* = 1.43; *SD* = 1.24) scales, whereas Perspicuity was rated excellent (*M* = 2.05; *SD* = 0.86) (see Fig. 4). The other scales were rated above average: Efficiency (*M* = 1.30; *SD* = 0.90), Dependability (*M* = 1.40; *SD* = 0.85), and Novelty (*M* = 0.84; *SD* = 1.24).

### CCT program group differences in UX (iCCT vs. bCCT)

The Perspicuity scale showed significantly higher values for the bCCT than for the iCCT, with a medium effect size for the group difference (Cohen’s *d* = −0.38, *p* =.006). For the other scales, small effect sizes (Cohen’s *d* < 0.2) and no significant (*p* >.05) differences between the two groups were detected (see Table 1).

**Table 1:** Group differences in the UX scales

UEQ dimension	M (SD)		t-Test	*p*	Cohen’s d	95%-CI
	*iCCT*	*bCCT*				
Attractiveness	1.64 (0.95)	1.73 (1.10)	−0.672	0.503	− 0.091	[−0.357;0.175]
Efficiency	1.21 (0.93)	1.30 (0.90)	−0.742	0.459	− 0.101	[−0.367;0.166]
Perspicuity	1.71 (0.90)	2.05 (0.86)	−2.779	0.006	− 0.377	[−0.645;−0.108]
Dependability	1.33 (0.88)	1.40 (0.85)	−0.578	0.564	− 0.078	[−0.345;0.188]
Stimulation	1.57 (0.98)	1.43 (1.24)	0.880	0.380	0.119	[−0.147;0.386]
Novelty	0.83 (1.15)	0.84 (1.24)	−0.061	0.951	− 0.008	[−0.274;0.258]

*Note. iCCT * individualized computerized cognitive training, *bCCT* basic computerized cognitive training, *CI * Confidence Interval of Cohen’s *d*

### Correlations between self-reported training intensity and the UX scales

There was no multicollinearity between self-reported training intensity and the UX scales in either of the two CCT programs (*r* <.8).

The intensity of the training was positively correlated with four UEQ scales for each CCT program. The following significant Pearson coefficients were found for the iCCT: Attractiveness *r* =.246 (*p* =.010), Perspicuity *r* =.248 (*p* =.009), Stimulation *r* =.201 (*p* =.036), and Novelty *r* =.223 (*p* =.020). Efficiency and Dependability were not significantly correlated with intensity. For the bCCT, the following positive correlations with intensity were found: Attractiveness *r* =.338 (*p* <.001), Efficiency *r* =.338 (*p* <.001), Stimulation *r* =.427 (*p* <.001), and Novelty *r* =.298 (*p* =.002). However, Perspicuity and Dependability were not significantly correlated with intensity.

### Sensitivity analysis

Concerning the iCCT, significant gender differences and moderate effect sizes were detected for Attractiveness, Dependability and Stimulation (see supplement material 3) such that women rated these three scales higher than men. Furthermore, Dependability showed significantly higher values for participants younger than 70 years, with a medium effect size (Cohen’s *d* = −0.55, *p* =.006).

For the bCCT, no significant gender differences and lower effect sizes were found, but multiple differences between the two age groups were identified. Participants aged 70 years or older gave higher ratings on Stimulation and Novelty (Cohen’s *d* = 0.44, *p* =.023; Cohen’s *d* = 0.59, *p* =.003, respectively), whereas participants younger than 70 years gave higher ratings on Dependability and Perspicuity (Cohen’s *d* = −0.68, *p* <.001; Cohen’s *d* = 0.49, *p* =.012, respectively).

## Discussion

The aim of the present analysis was to examine and compare UX for the two CCT programs from the BrainFit-Nutrition study, based on the benchmark of the UEQ [40]. Furthermore, we examined the relationship between self-reported training intensity and UX. In addition, we examined gender- and age-related differences in UX for the two CCT programs.

The results of the analyses showed that both CCT programs are user-friendly tools for the group of participants with MCI 60 years of age and older. From the users’ point of view, the CCT programs are well-suited for daily use, as the participants were satisfied overall. This finding is in line with previous CCT studies that have also found satisfaction with CCT programs among people with cognitive impairment [25, 26]. Satisfaction is also an important aspect for the intentions of people with MCI to continue using the CCT [25]. According to Contreras-Somoza et al. [19], the user-friendliness of the two CCT programs is a key aspect - alongside appropriateness regarding skills and knowledge - for people with MCI to benefit from CCTs [19, 43]. The two CCT programs therefore also provide needs-appropriate support for people with MCI [19]. However, it should not be neglected that the sample consisted of a high proportion of highly educated and digitally skilled participants compared to the average population in this age cohort, which may limit the generalizability of the results.

According to the UEQ benchmarking [40], both CCT programs fulfilled user expectations. Overall, both CCT programs exhibited satisfactory performance on all scales, and no scales were below average. However, the two scales Efficiency and Dependability (both aspects of pragmatic quality) were less reliable, whereby the Dependability scale already showed inconsistencies in a small percentage of the sample in Contreras-Somoza et al. [41]. Even though the questionnaire is considered suitable for recording the impressions of these patient groups regarding technological tools [41]. According to the UEQ-handbook [43], the wording of the items is not completely without misinterpretations, especially in older adults or populations with cognitive impairment. However, comprehension problems do not necessarily stem directly from cognitive decline, as even computer science students can misunderstand or misinterpret certain UEQ questions [41]. Thus, the low reliability seems to indicate less a weakness in the scales than rather comprehension problems in the study population. However, the other four scales had good or even very good reliability. A comparison of the UX of the two CCT programs in the study showed that there were no significant differences in UX between the two forms of CCT program, except for Perspicuity, such that participants rated the bCCT tending to as more comprehensible than the iCCT. A possible explanation for this could be that the bCCT included fewer exercises than the iCCT, allowing participants to familiarize themselves with the bCCT a little better. Thus, the bCCT appears to be more intuitive for the target group of individuals with MCI 60 years of age or older, which is important for avoiding frustration during training [22]. However, due to its simplicity, there is a risk that the bCCT may not be challenging enough for people with MCI to achieve improvements in global cognition. This should be further investigated in future studies.

In comparison to the iCCT, the bCCT, which was the program given to the active control group, was cognitively less challenging for the participants. Therefore, the higher demands placed on the iCCT participants tended to lead to limited comprehensibility, because they had to learn how to use the iCCT system. However, this result contradicts the findings of the pilot study, which found better UX results for the iCCT group than for the bCCT group [37].

Furthermore, satisfaction with particular aspects was associated with self-reported training intensity. This finding shows the consistency of our data because the participants who tend to use the program more frequently are more likely to be satisfied with particular aspects of the program. For both CCT programs, Attractiveness, Novelty, and Stimulation were significantly but moderately correlated with training intensity. These findings indicate that when users appreciated the hedonic quality (Novelty and Stimulation) of the CCT program, they used it more frequently. A higher training intensity can lead to long-term adherence to training due to the positive hedonic aspects and a resulting positive UX. Thereby, a longer training period for people with neurodegenerative diseases is associated with an increase in perceived competence and interest [29]. This finding is in line with the finding by Schrepp et al. [44] that also non-task-related quality aspects play important roles for users. However, the duration of training does not necessarily correspond to satisfaction with the program [25].

The increased training intensity is also an indicator of the intrinsic motivation of CCT program users to improve their cognitive performance by increasing the frequency or duration of training [26]. This intrinsic motivation to use a CCT program also goes along with potential reasons for participating in the study, such as improving cognitive health [22, 27] and avoiding cognitive decline [28]. This allows participants to fulfill their need for autonomy and experience of competence, which increases intrinsic motivation according to self-determination theory [45, 46]. The present results indicate that both the iCCT and the bCCT satisfy these needs. In addition, intrinsic motivation can also potentially be attributed to the enjoyment of the program [22, 24]. As mentioned above, the UX aspect of hedonic quality (Stimulation and Novelty) had an influence on training intensity for both CCT programs. Moreover, the participants using bCCT engaged in exercising their brains more frequently when they were satisfied with the Efficiency, whereas the participants using iCCT did not. For them, in turn, Perspicuity was significant. In sum, the overall dimension pragmatic quality did not appear to influence training intensity in both CCT groups. According to the sensitivity analysis, approval ratings depended first on whether women or men used the CCT program. Second, participants’ age made a difference. The iCCT worked better for women than for men, especially regarding Attractiveness, Dependability, and Stimulation. Thus, while using the iCCT women appear to be more satisfied than men. No age differences were found for the iCCT except for the Dependability scale. Accordingly, the younger cohort found the iCCT to be more reliable and predictable than the older cohort. Maybe, the iCCT is too challenging for people aged 70 years and older in terms of Dependability. For the bCCT, no gender differences were found. Furthermore, the bCCT performed better for the older cohort regarding the hedonic aspects of the tool. For the younger cohort, in turn, the pragmatic aspects worked better. This is because the extent of age-related perceived hedonic and pragmatic quality can be influenced by digital literacy, and technology familiarity, which is low in today’s Silent and Boomer generation, who are digital immigrants, relative to digital natives. Especially in combination with the individual cognitive processing demands, which depend on the current cognitive decline during MCI, these two quality scales can differ various along the participants’ age.

The aspects mentioned in the literature, such as problems with the user interface of the CCT program [30] or perceived competence problems in dealing with the technology [22, 29] could not be verified in the present analysis.

### Strengths and limitations

This study has some strengths and limitations. As limitations, first, the conclusiveness of the UX results in relation to the CCT programs was limited by the fact that one fifth of the participants did not respond, which can lead to a bias in the UX results of the two CCTs, including an over- or underestimation. Second, the sample was biased by participants’ high educational level and willingness to use digital tools. Third, the Efficiency and Dependability scales had low reliabilities, which limits the validity of the results of these scales. Fourth, judgment biases such as social desirability cannot be ruled out for some variables, as these were only collected as self-reports. In particular, training intensity was not tracked objectively using the CCT program, which will allow better conclusions about the optimal dose-response relationship to be made in the future. Fifth, the program did not monitor the input method and the number of sessions dedicated by the participants.

However, the study also had some strengths. First, despite the use of secondary data from a randomized controlled study, the present sample was quite large compared with previous studies. Second, not only did we analyze the differences between the two CCT programs, but we also performed sensitivity analyses regarding age and gender differences. Thus, we conducted a comprehensive analysis of the UX of the two CCT programs.

## Conclusion

Both CCT programs showed overall a good UX. However, there were no significant UX differences between iCCT and bCCT in 5 of the 6 UEQ scales. Merely the Perspicuity scale showed significantly higher values for the bCCT than for the iCCT, so the higher requirements of the iCCT program led probably to limitations in comprehensibility. Participants who were satisfied with the aspects Attractiveness and hedonic quality (Stimulation and Novelty) were using both CCT programs more intensely. For an optimal experience of users ≥ 70 or lower digital literacy, they could profit from an onboard with the bCCT, and then possibly scaffold to iCCT.

## Supplementary Information

Supplementary Material 1

Supplementary Material 2

Supplementary Material 3

## Data Availability

The data presented in this study are available upon reasonable request from the corresponding author. The data are not publicly available due to privacy.

## Abbreviations

CCT: Computerized Cognitive Training
bCCT: Basic Computerized Cognitive Training
iCCT: Individualized Computerized Cognitive Training
ISCED: International Standard Classification of Education
MCI: Mild Cognitive Impairment
UEQ: User Experience Questionnaire
UX: User Experience
