# Supplementary material for: User experience with two computerized cognitive intervention programs for people with mild cognitive impairment
Source: BMC Geriatr. 2025 Dec 10;26:29. doi: 10.1186/s12877-025-06767-y (PMC12784485; doi:10.1186/s12877-025-06767-y)
Supplement: Supplementary file 1 — Supplementary Material 1 [file 12877_2025_6767_MOESM1_ESM.docx]

**Supplement Material 2**. *Reliabilities of the UX Dimensions*

|  | **Reliability** | | | | | |
| --- | --- | --- | --- | --- | --- | --- |
|  | Attractiveness | Efficiency | Perspicuity | Dependability | Stimulation | Novelty |
| iCCT (*n* = 109) | .88 | .52 | .65 | .50 | .76 | .74 |
| bCCT (*n* = 108) | .90 | .50 | .65 | .40 | .87 | .77 |

*Note*. Reliabilities were measured with Cronbach’s α.
