# Supplementary material for: User experience with two computerized cognitive intervention programs for people with mild cognitive impairment
Source: BMC Geriatr. 2025 Dec 10;26:29. doi: 10.1186/s12877-025-06767-y (PMC12784485; doi:10.1186/s12877-025-06767-y)
Supplement: Supplementary file 2 — Supplementary Material 2 [file 12877_2025_6767_MOESM2_ESM.docx]

**Supplement material 3**. *Age and gender related group differences in the UX scales*

| **UEQ dimension** | ***M* (*SD*)** | | ***t-*Test** | ***p*** | **Cohen’s *d*** | **95%-*CI*** |
| --- | --- | --- | --- | --- | --- | --- |
| **Gender differences** | | | | | |  |
| *iCCT* | *Female (n = 57)* | *Male (n = 52)* |  |  |  |  |
| Attractiveness | 1.87 (0.87) | 1.38 (0.98) | 2.777 | .006 | .533 | [.149;.914] |
| Efficiency | 1.37 (0.91) | 1.02 (0.93) | 1.973 | .051 | .378 | [-.002;.757] |
| Perspicuity | 1.76 (0.84) | 1.66 (0.97) | 0.602 | .548 | .115 | [-.261;.491] |
| Dependability | 1.51 (0.91) | 1.13 (0.81) | 2.732 | .019 | .455 | [.073;.835] |
| Stimulation | 1.81 (0.97) | 1.30 (0.93) | 2.766 | .007 | .530 | [.147;.912] |
| Novelty | 0.99 (1.21) | 0.65 (1.07) | 1.526 | .128 | .295 | [-.084;.672] |
|  |  |  |  |  |  |  |
| *bCCT* | *Female (n = 57)* | *Male (n = 51)* |  |  |  |  |
| Attractiveness | 1.79 (1.15) | 1.67 (1.05) | 0.591 | .556 | .114 | [-.264;.492] |
| Efficiency | 1.28 (1.00) | 1.32 (0.77) | -0.272 | .786 | -.052 | [-.430;.326] |
| Perspicuity | 2.0 (0.94) | 2.10 (0.78) | -0.644 | .521 | -.124 | [-.502;.254] |
| Dependability | 1.42 (0.82) | 1.37 (0.89) | 0.296 | .768 | .057 | [-.321;.435] |
| Stimulation | 1.41 (1.35) | 1.46 (1.13) | -0.181 | .857 | -.035 | [-.413;.343] |
| Novelty | 0.76 (1.36) | 0.93 (1.11) | -0.718 | .475 | -.138 | [-.516;.240] |
|  |  |  |  |  |  |  |
| **Age differences** | | | | | |  |
| *iCCT* | *≥ 70 y. (n = 54)* | *< 70 y. (n = 55)* |  |  |  |  |
| Attractiveness | 1.61 (1.13) | 1.67 (0.75) | -0.335 | .738 | -.064 | [-.440;.311] |
| Efficiency | 1.05 (0.95) | 1.36 (0.90) | -1.738 | .085 | -.333 | [-.710;.046] |
| Perspicuity | 1.59 (0.93) | 1.83 (0.86) | -1.444 | .152 | -.277 | [-.653;.101] |
| Dependability | 1.10 (0.92) | 1.56 (0.79) | -2.823 | .006 | -.541 | [-.922;-.157] |
| Stimulation | 1.62 (0.92) | 1.51 (1.04) | -0.603 | .548 | -.116 | [-.491;.261] |
| Novelty | 0.88 (1.20) | 0.79 (1.10) | 0.400 | .690 | .077 | [-.299;.452] |
|  |  |  |  |  |  |  |
| *bCCT* | *≥ 70 y. (n = 54)* | *< 70 y. (n = 54)* |  |  |  |  |
| Attractiveness | 1.78 (1.04) | 1.69 (1.17) | 0.421 | .675 | .081 | [-.297;458] |
| Efficiency | 1.31 (0.87) | 1.28 (0.93) | 0.187 | .852 | .036 | [-.341;.413] |
| Perspicuity | 1.77 (0.94) | 2.32 (0.68) | -3.528 | <.001 | -.678 | [-1.064;-.288] |
| Dependability | 1.19 (0.77) | 1.60 (0.88) | -2.562 | .012 | -.493 | [-.875;-.109] |
| Stimulation | 1.70 (1.12) | 1.16 (1.31) | 2.309 | .023 | .444 | [.061;.825] |
| Novelty | 1.19 (0.95) | 0.49 (1.41) | 3.071 | .003 | .591 | [.204;.975] |

*Note*. iCCT = individualized computerized cognitive training; bCCT = basic computerized cognitive training. CI = Confidence Interval of Cohen’s *d*.
