# Supplementary material for: User experience with two computerized cognitive intervention programs for people with mild cognitive impairment
Source: BMC Geriatr. 2025 Dec 10;26:29. doi: 10.1186/s12877-025-06767-y (PMC12784485; doi:10.1186/s12877-025-06767-y)
Supplement: Supplementary file 3 — Supplementary Material 3 [file 12877_2025_6767_MOESM3_ESM.docx]

**Supplement material 1. Sample images of the computerised cognitive training programs in the BrainFit-Nutrition study**

1. Inidividualised computerised cognitive training (iCCT)

Choose an exercise!


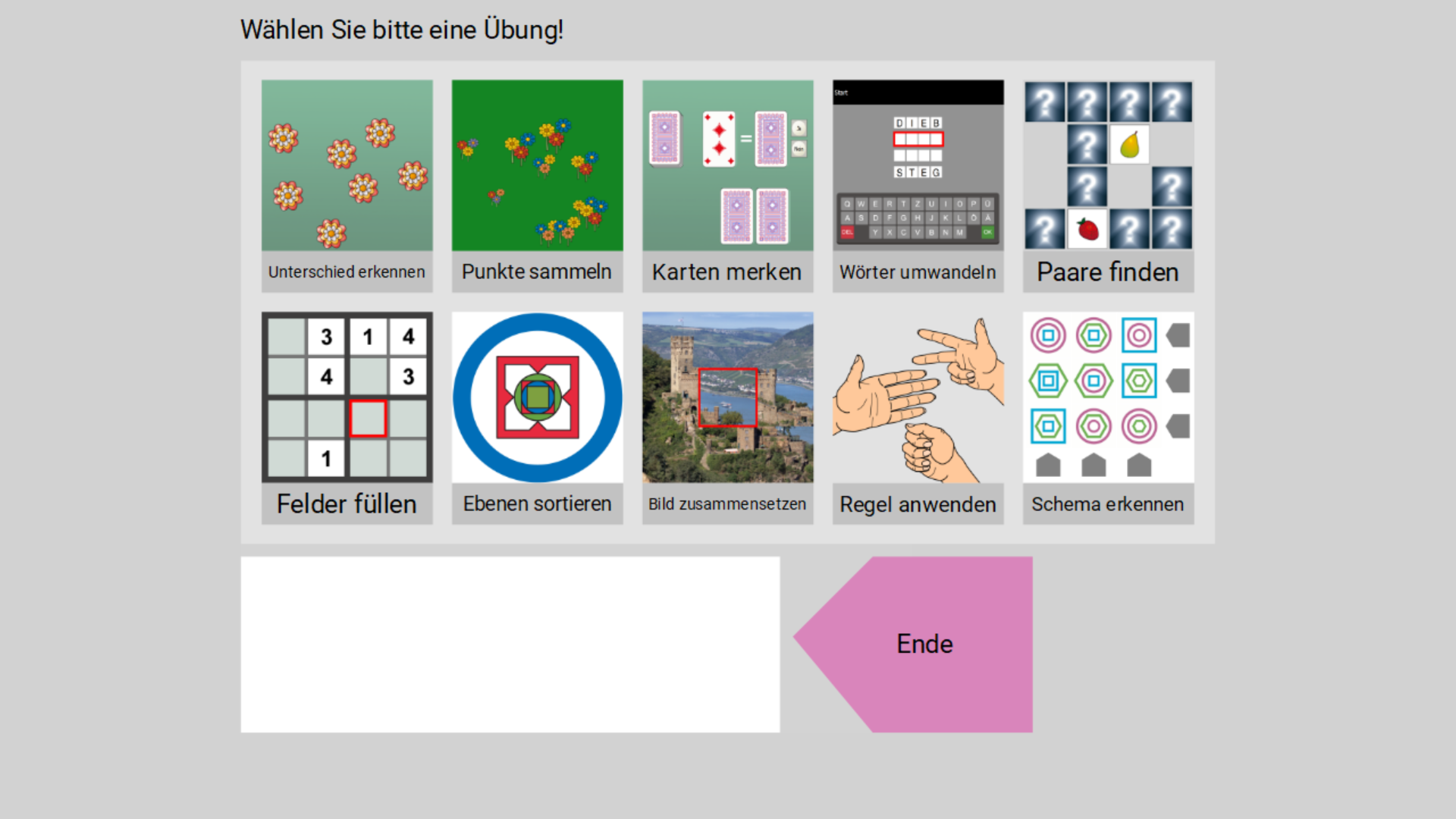


Pattern recognition

Applying rules

Jigsaw puzzle

Layer sorting

Fill in the gaps

Find pairs

Word conversion

Remember cards

Finding targets

Spot the difference


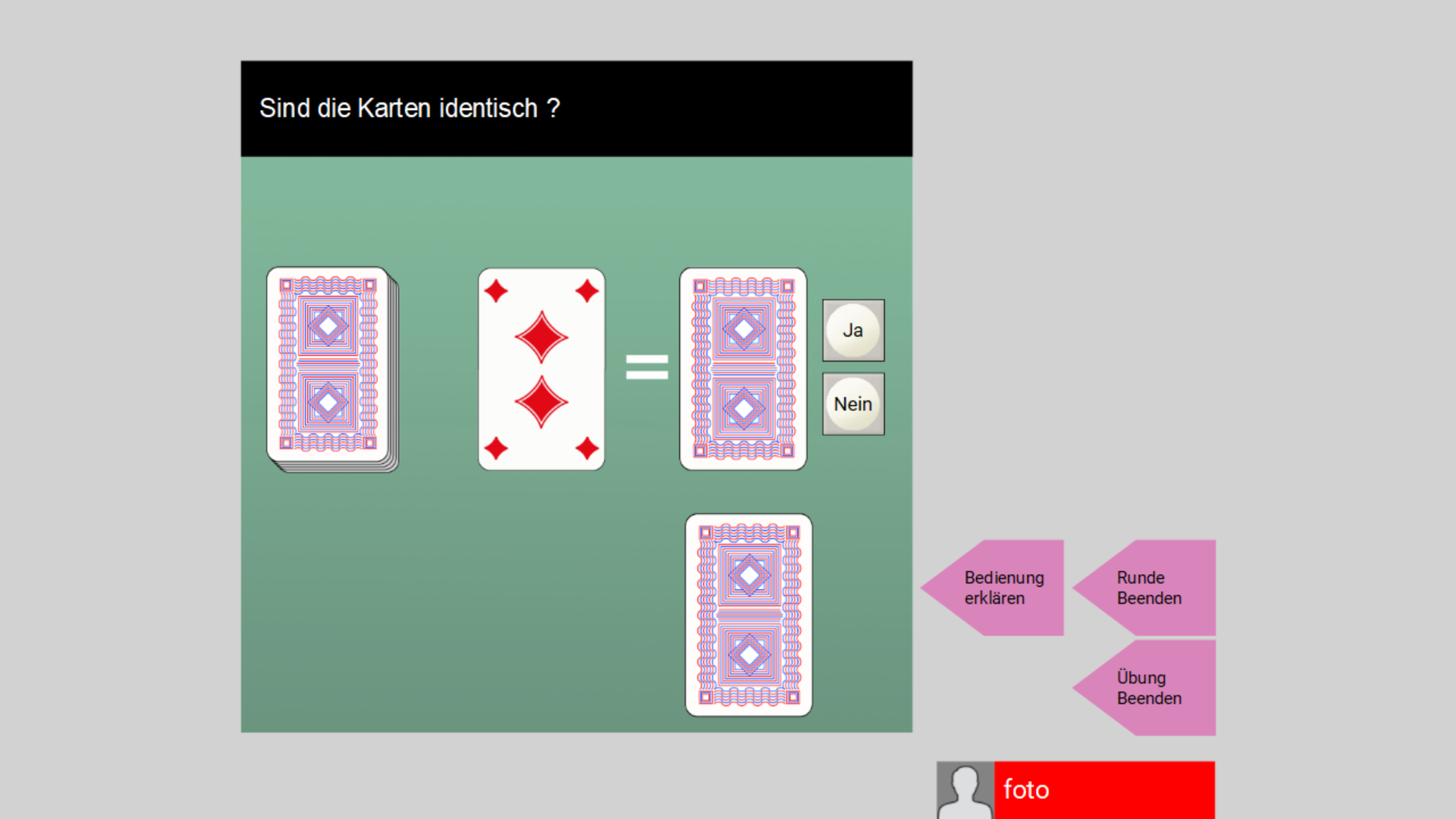


Are the cards the same?

1. Basic computerised cognitive training (bCCT)


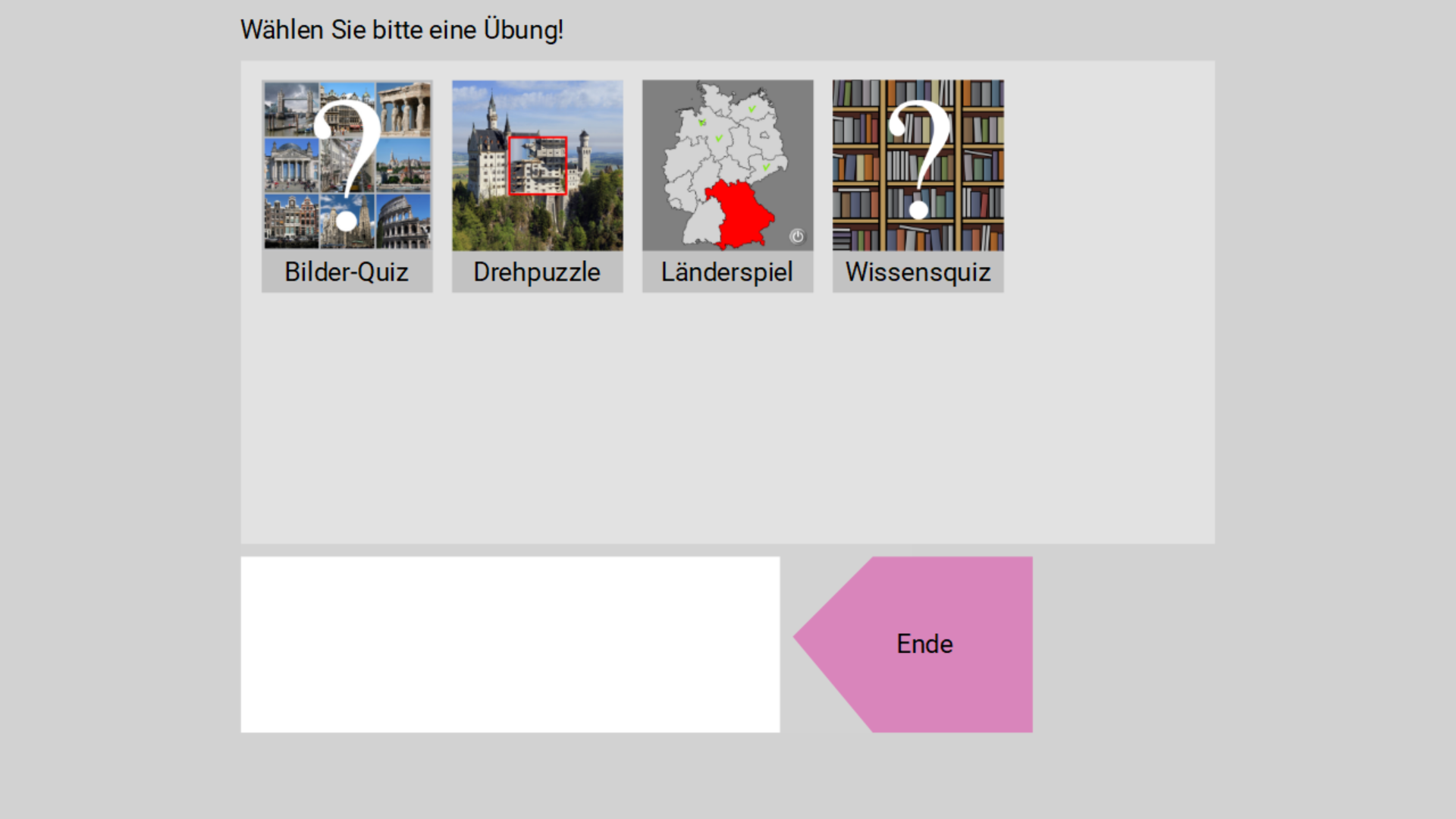


Quiz-show

Geography quiz

Rotating picture puzzle

Choose an exercise!


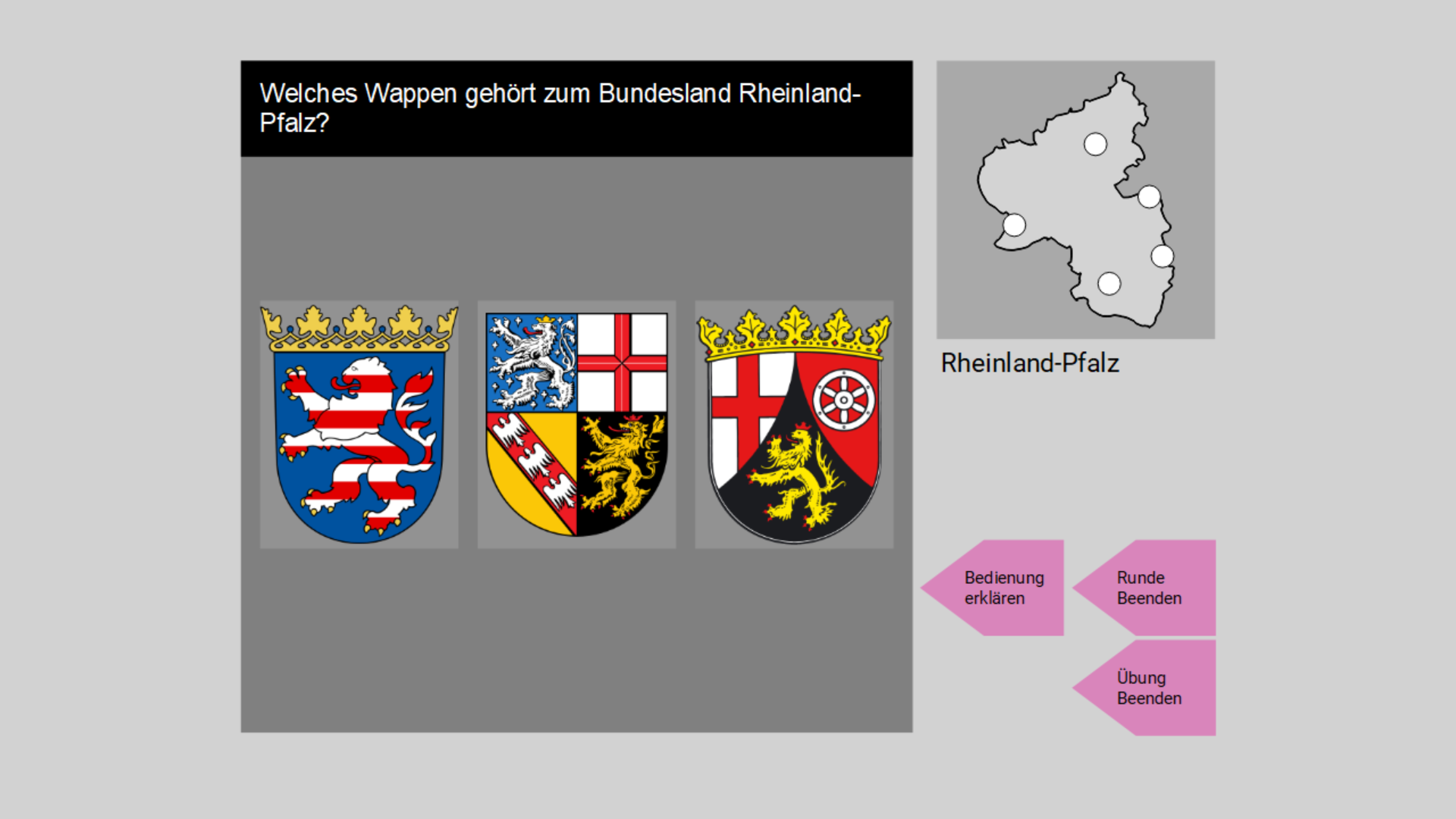


which emblem belongs to the state of Rheinland-Pfalz?
